# Supplementary figures and images for: Novel reporter of the PINK1-Parkin mitophagy pathway identifies its damage sensor in the import gate
Source: bioRxiv. 2025 Feb 20:2025.02.19.639160. Preprint. [Version 1] doi: 10.1101/2025.02.19.639160 (PMC11870511; doi:10.1101/2025.02.19.639160)

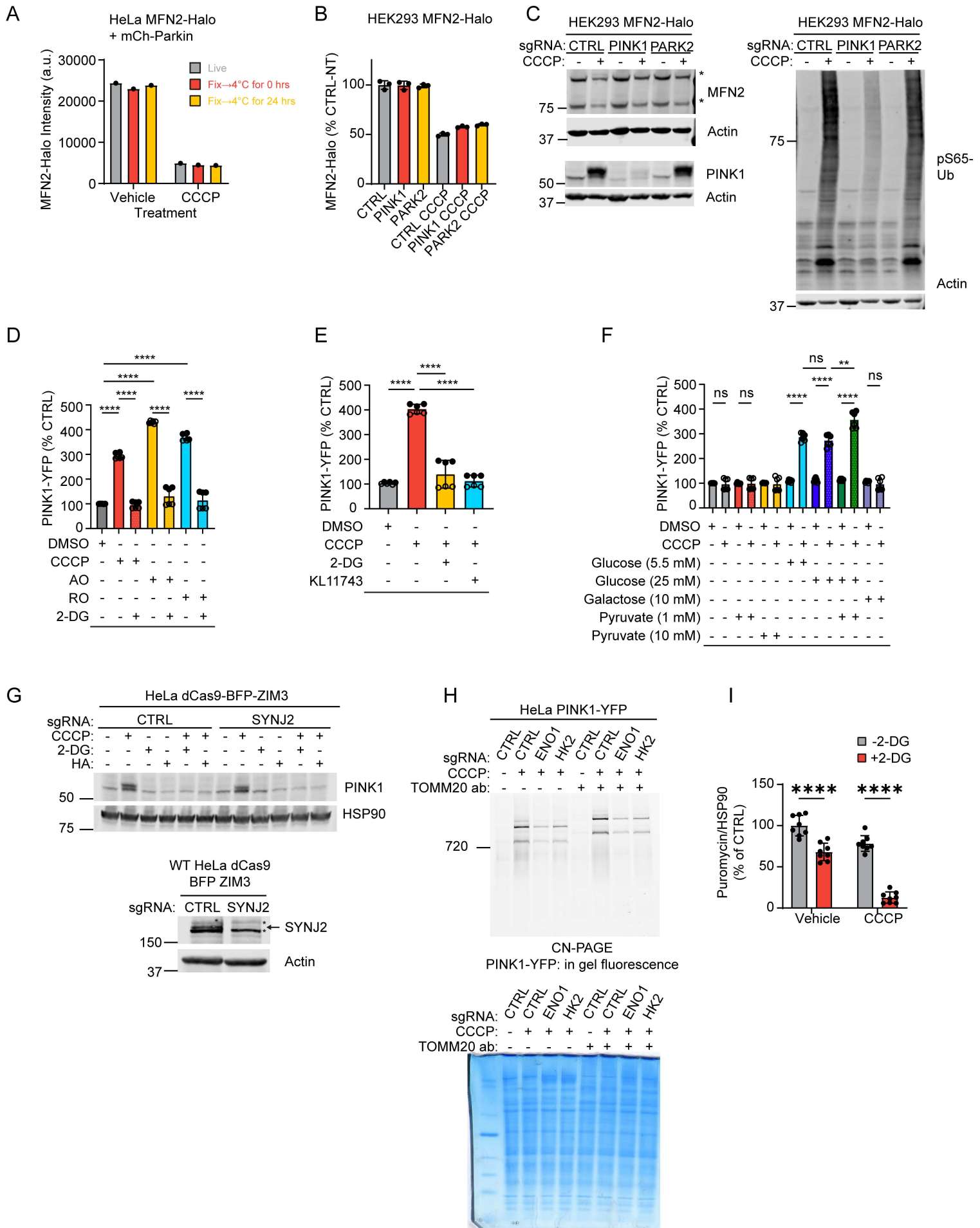

Figure S2

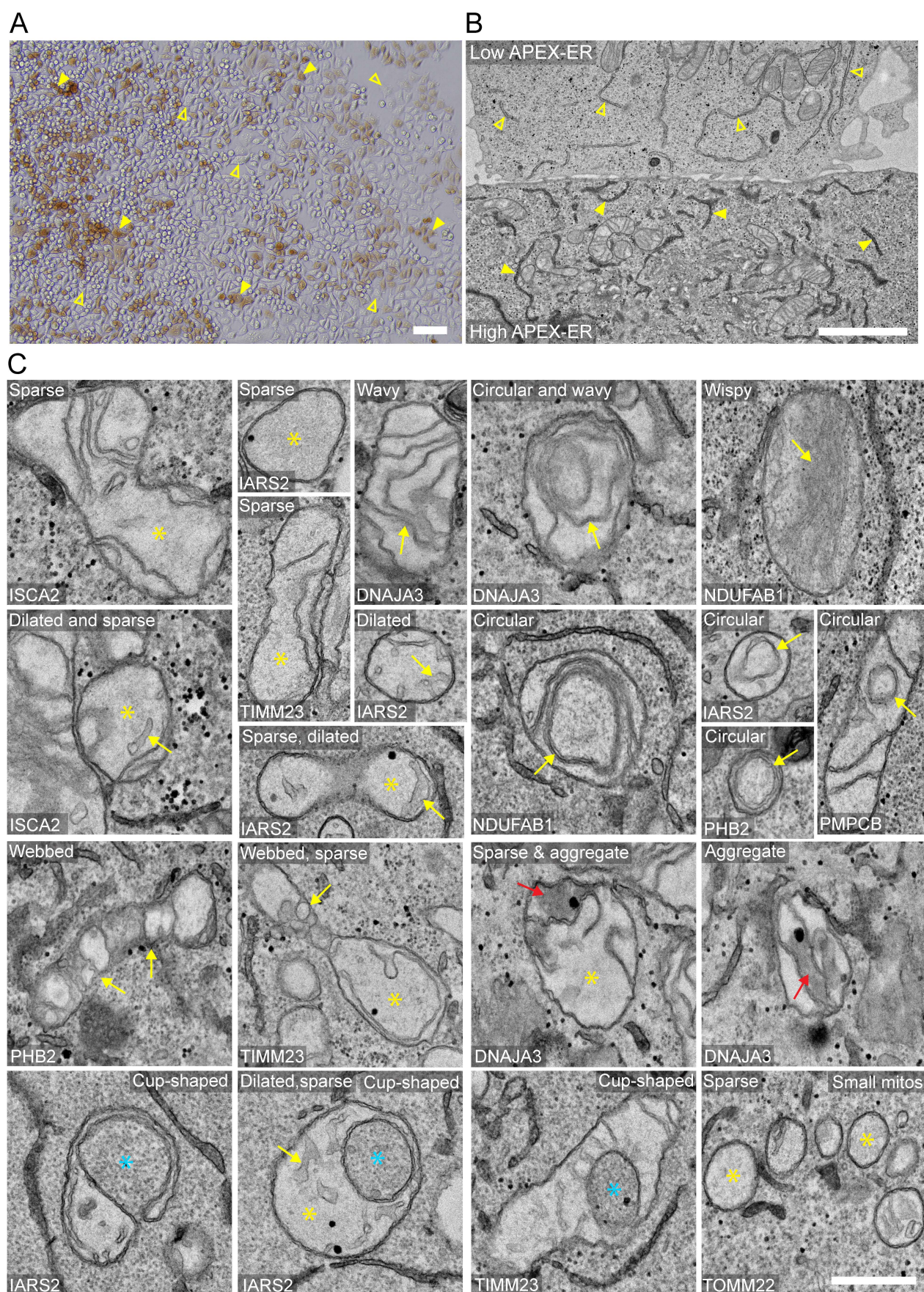

# Figure 3

**A**

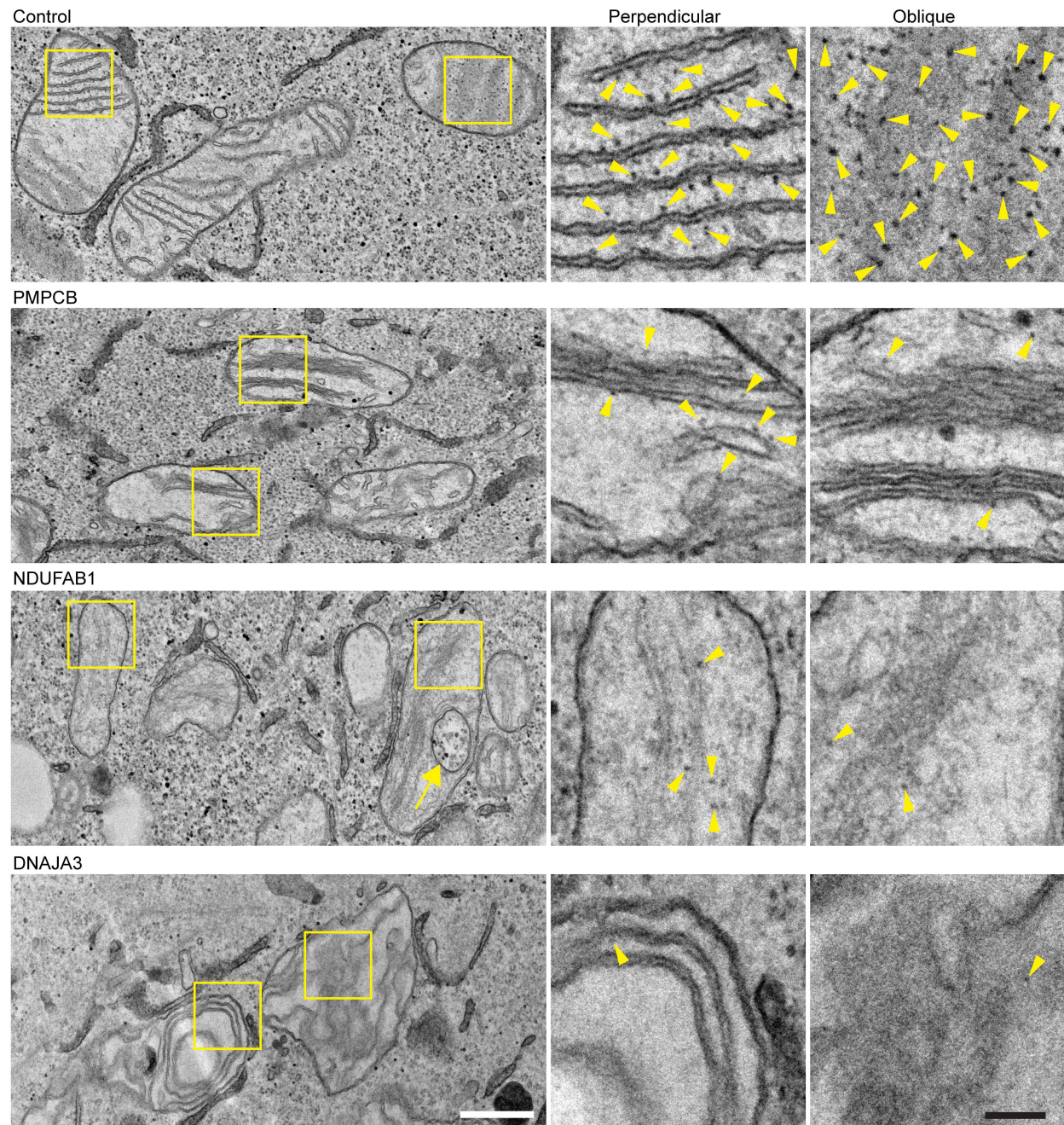

**B**

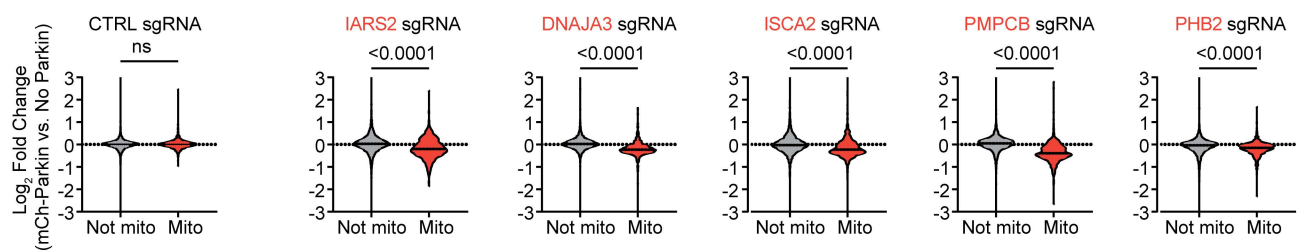

Figure S4

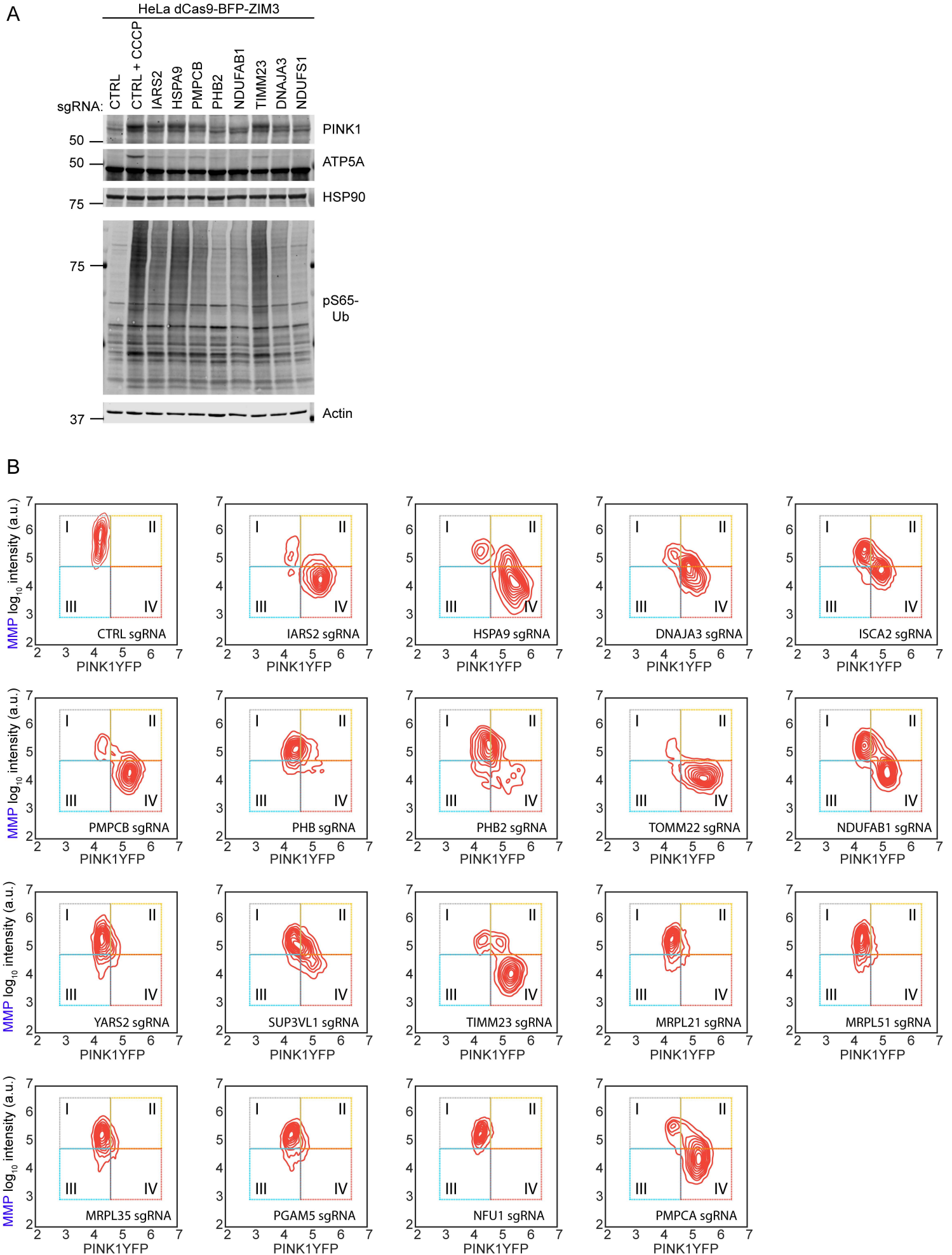

Figure S5

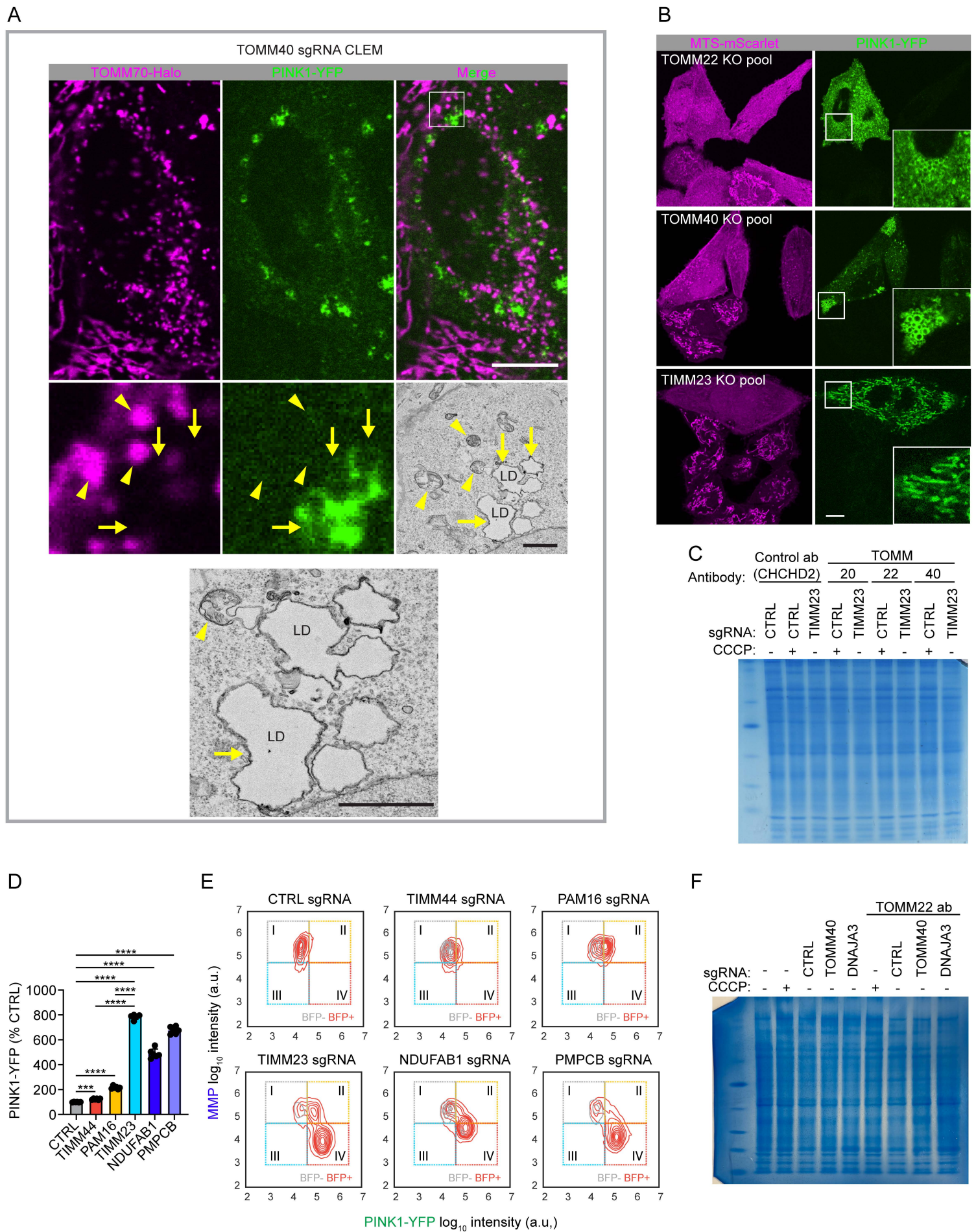

Supplement: 1 — Supplemental Figure 1. MFN2-HaLo reporter validation and glycolysis is required for PINK1-YFP stabilization following OXPHOS impairment. (A) Flow cytometry of HeLaMFN2-Halo+mCh-Parkin cells +/− 10 μM CCCP 4hrs with or without fixation. N = 1 independent experiment. (B) Flow cytometry of HEK293MFN2-Halo cells +/− 10 μM CCCP O/N. N = 3 replicates on 1 occasion. (C) Representative immunoblots of endogenously tagged and untagged MFN2 alleles (top and bottom band, respectively) +/− 10 μM CCCP 4 hrs from HEK293MFN2-Halo cells. * denotes ubiquitinated MFN2 band. N = 2 replicates on 1 occasion. (D) Flow cytometry measurements in HeLaPINK-YFP cells treated with 10 μM CCCP, and 8 μg/mL antimycin + 10 μg/mL oligomycin, 1 μM rotenone + 10 μg/mL oligomycin, and/or 10 mM 2-DG, for 4 hrs. **** p ≤ 0.0001 Error bars mean +/− SD. N = 6 independent experiments from 2 separate transductions. (E) Flow cytometry measurements in HeLaPINK1-YFP cells treated with 10 μM CCCP, 10 μM KL11743 and/or 10 mM 2-DG, for 4 hrs. **** p ≤ 0.0001 Error bars mean +/− SD. N = 6 independent experiments from 2 separate transductions. (F) Flow cytometry measurements in HeLaPINK1-YFP cells treated with 10 μM CCCP +/− in glucose and pyruvate-free DMEM supplemented with glucose, galactose, and/or pyruvate at the indicated concentrations for 4 hrs. **** p ≤ 0.0001 Error bars mean +/− SD. N = 6 independent experiments from 2 separate transductions. (G) Representative immunoblots of HeLadCas9-BFP-ZIM3 cells treated with 10 μM CCCP, 10 μM HA, and/or 10 mM 2-DG 4 hrs. * denotes non-specific bands, arrow denotes position of SYNJ2 band. N = 2 independent experiments. (H) CN-PAGE separated PINK1-YFP complexes visualized by in gel fluorescence as in (Fig. 5J) (top) and total protein measured via SimplyBlue SafeStain (bottom). HeLa cells were cultured with indicated sgRNA for at least 7 days before CN-PAGE sample collection. N = 3 replicates from at least 2 transductions. (I) Quantification of immunoblots represented in [file NIHPP2025.02.19.639160V1-supplement-1.pdf]
